# DDAF-Net: Decoupled and Differentiated Attention Fusion Network for Object Detection

**DOI:** 10.3390/s26061812

**Published:** 2026-03-13

**Authors:** Bo Yu, Guanghui Zhang, Qun Wang, Lei Wang

**Affiliations:** 1 Shenyang Institute of Computing Technology, Chinese Academy of Sciences, Shenyang 110168, China; yubo@sict.ac.cn (B.Y.); wangqun@sict.ac.cn (Q.W.); 2 University of Chinese Academy of Sciences, Beijing 100049, China; 3 China Coal Technology and Engineering Robotics Technology Co., Ltd., Shenzhen 518000, China; wincoat@163.com; 4 China Coal Technology and Engineering (Liaoning) Embodied Intelligent Technology Co., Ltd., Shenyang 110000, China

**Keywords:** multimodal sensor fusion, object detection, feature decoupling, attention mechanism, gated fusion

## Abstract

**Highlights:**

**What are the main findings?**

- A decoupled RGB–IR framework explicitly separates modality-common and -specific features, minimizing redundancy while retaining complementary details.
- A differentiated attention strategy robustly aligns cross-modal semantics and suppresses noise, achieving state-of-the-art performance on LLVIP and M^3^FD datasets.

**What are the implications of the main findings?**

- The proposed “decoupling–enhancement–fusion” paradigm offers a robust solution to modality redundancy, spatial misalignment, and noise interference.
- Its lightweight attention and adaptive gating mechanisms provide an efficient design extensible to other heterogeneous sensor fusion tasks.

**Abstract:**

The fusion of data from visible (RGB) and infrared (IR) sensors is essential for robust all-day and all-weather object detection. However, existing methods often suffer from modality redundancy and noise interference. To address these challenges, we propose the Decoupled and Differentiated Attention Fusion Network (DDAF-Net). Architecturally, DDAF-Net employs a decoupled backbone with a Siamese weight-sharing strategy to extract modality-common features, while parallel branches capture modality-specific features. To effectively integrate these features, we design the Differentiated Attention Fusion Module (DAFM). First, we introduce Spatial Residual Unshuffle Embedding (SRUE) to achieve lossless downsampling while preserving global semantic information. Second, differentiated attention mechanisms are applied for feature enhancement: Dual-Norm Alignment Attention (DNAA) facilitates effective modal alignment and enhances semantic consistency in modality-common features, while Sparse Purification Attention (SPA) enables selective utilization of complementary information by suppressing noise and focusing on salient regions in modality-specific features. Finally, the Adaptive Complementary Fusion Module (ACFM) integrates these components by using modality-common features as a baseline and dynamically weighting the complementary modality-specific information. Extensive experiments on public datasets such as LLVIP and M^3^FD demonstrate that DDAF-Net achieves state-of-the-art performance. These results validate the effectiveness of our proposed decoupling–enhancement–fusion paradigm.

## 1. Introduction

Multimodal fusion aims to integrate information from diverse sensors to achieve environmental perception capabilities that are more comprehensive and robust than those of single-modality systems. This technology has demonstrated significant advantages in various fields, including autonomous driving [1], industrial defect inspection [2], medical image analysis [3], and emotion recognition [4]. In object detection tasks, a single sensor often struggles to maintain stable performance within complex and dynamic environments. Visible (RGB) images provide rich color and texture information, facilitating precise object localization and category discrimination; however, their performance deteriorates significantly under low-light, high-glare, or adverse weather conditions. In contrast, infrared (IR) images provide stable thermal radiation contours in low light or total darkness but typically lack fine-grained textural details and high-level semantic representation. Consequently, fusing RGB and IR images—two heterogeneous modalities with significant complementarity—is widely regarded as an effective approach for achieving robust all-day and all-weather object detection.

**Figure 1:**
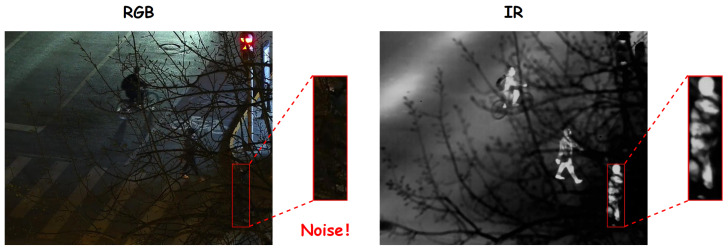
Illustration of modality-complementarity and modality-specific noise in paired RGB–IR images under nighttime occlusion.

Despite the immense potential of RGB-IR multimodal fusion in object detection, existing methods still face several key challenges during the modeling process. First, modality redundancy is prevalent. Different sensors often contain substantial semantically overlapping common information [5] (e.g., geometric structures and contour shapes) when describing the same object. Without explicit constraints during the feature extraction or fusion stage, this redundancy leads to repetitive modeling, which not only increases computational overhead but may also weaken discriminative semantic expression. Second, modality-specific information, while complementary, inevitably introduces noise interference. As illustrated in Figure 1, in nighttime scenarios, the RGB modality often contains significant background noise due to poor illumination or occlusion, whereas the IR modality preserves clear object contours. If such modality-specific noise is not effectively suppressed during fusion, unreliable RGB responses may be erroneously amplified and introduced into the fused features, thereby interfering with discriminative representation and degrading detection robustness. Third, modality misalignment remains a prominent issue. Due to differences in sensor imaging mechanisms, viewpoint offsets, and resolutions, RGB and IR features often exhibit deviations in spatial layout and semantic response, hindering the establishment of cross-modal semantic consistency. Finally, fusion strategy designs remain relatively coarse. A significant number of existing methods rely on simple feature concatenation or element-wise addition, lacking explicit fusion guidance mechanisms. This makes it difficult to model complex cross-modal dependencies, often leading to modality dominance or information suppression.

To address these issues, we propose the Decoupled and Differentiated Attention Fusion Network (DDAF-Net), which resolves these challenges through a targeted modular design. To tackle modality redundancy, DDAF-Net introduces a Siamese weight-sharing strategy [6,7] at the backbone level, explicitly decoupling RGB and IR features into modality-common features and modality-specific features. The weight-sharing branch learns cross-modal invariant semantics by enforcing parameter consistency, effectively avoiding repetitive modeling of common information, while parallel modality-specific branches retain the discriminative expression capabilities of each modality. On this basis, we design the Differentiated Attention Fusion Module (DAFM) to further achieve feature enhancement and cross-modal alignment. First, the Spatial Residual Unshuffle Embedding (SRUE) is introduced to perform lossless downsampling while effectively preserving global contextual semantics. Subsequently, to address noise interference in modality-specific features, we introduce Sparse Purification Attention (SPA). By utilizing a sparse attention mechanism to suppress non-salient responses and focus on key regions, SPA enables the selective utilization of complementary information, thereby improving feature robustness. Regarding modality misalignment, we propose Dual-Norm Alignment Attention (DNAA), which utilizes Sinkhorn iterations to explicitly establish correspondences between RGB and IR features at both channel and spatial levels, promoting semantic consistency modeling of modality-common features. Finally, to overcome the lack of effective guidance in fusion strategies, we propose the Adaptive Complementary Fusion Module (ACFM). This module uses the aligned modality-common features as a semantic anchor, dynamically generating gating weights to adaptively regulate the fusion ratio of modality-specific information, thus achieving stable and complementary cross-modal feature integration.

Based on the above design, the main contributions of this paper are summarized as follows:

1. We propose an RGB-IR fusion framework with explicit specificity–commonality decoupling, effectively mitigating modality redundancy and cross-modal interference through a Siamese weight-sharing strategy.
2. We design the DAFM, which combines DNAA and SPA to achieve semantic alignment of modality-common features and noise suppression of modality-specific features, respectively.
3. We propose the ACFM, which establishes a dynamic weighting mechanism using modality-common features as a semantic anchor, effectively balancing the fusion of cross-modal complementary information across varying scenarios.
4. Extensive experiments on public datasets such as LLVIP and M^3^FD demonstrate that the proposed DDAF-Net achieves performance superior to existing methods in RGB-IR object detection tasks.

The remainder of this paper is organized as follows. Section 2 reviews related work. Section 3 presents the overall architecture and core modules of DDAF-Net. Section 4 provides experimental results and a detailed analysis. Finally, Section 5 concludes the paper and discusses future research directions.

## 2. Related Work

Research on multimodal fusion can generally be categorized along two dimensions: fusion stage and fusion mechanism. The fusion stage focuses on where multimodal interactions occur within a network, while the fusion mechanism concerns how these interactions and integrations are realized. This section reviews representative studies from both perspectives.

### 2.1. Fusion Stages

**Figure 2:**
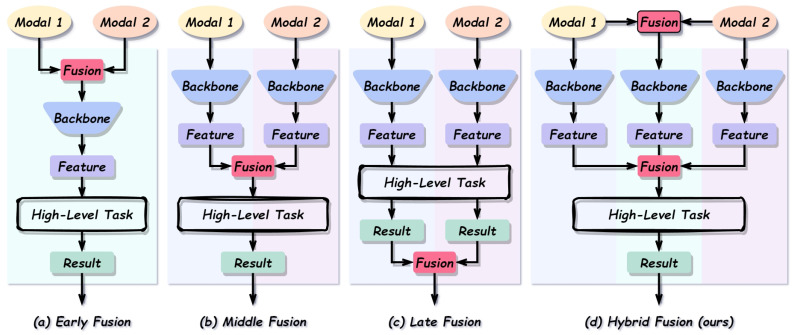
Comparison of early, middle, late, and hybrid fusion paradigms.

According to the position at which multimodal information is integrated, existing methods are commonly divided into early fusion, middle fusion, late fusion, and hybrid fusion paradigms [8], as illustrated in Figure 2.

Early fusion integrates multimodal information directly at the data or shallow feature level [9]. This paradigm is simple and preserves fine-grained raw information; however, it is highly sensitive to modality noise or missing data, and naive feature concatenation often leads to the curse of dimensionality. Middle fusion performs cross-modality interaction at intermediate layers [10], enabling semantic alignment and complementarity between modalities. However, its effectiveness heavily depends on the choice of fusion layer(s) and the overall network design. Late fusion processes each modality independently and combines the predictions at the decision stage using strategies such as voting, weighted averaging, or ensemble methods [11]. Although this paradigm offers strong modularity and robustness, the absence of intermediate feature interaction prevents the effective exploitation of complementary mid-level representations. Hybrid fusion combines multiple fusion stages by introducing cross-modal interactions at different network depths [12]. While this paradigm achieves greater flexibility and often superior performance, it also increases model complexity and computational overhead, and may still suffer from redundant feature modeling or insufficient guidance for selectively utilizing modality-specific complementary information.

### 2.2. Fusion Mechanisms

Fusion mechanisms determine how multimodal features interact and are integrated within a network, thereby directly influencing both representational capacity and computational efficiency. Based on their underlying principles, existing fusion mechanisms can be broadly categorized as follows.

Operator-based fusion combines multimodal features using simple mathematical operations such as concatenation, weighted summation, or element-wise multiplication [13]. These approaches are computationally efficient and easy to implement; however, their limited modeling capacity makes it difficult to capture complex cross-modal dependencies.

Attention-based fusion has been widely adopted to model both intra-modal contextual relationships and inter-modal interactions [14,15]. Self-attention enhances contextual representation within each modality [16,17], while cross-attention facilitates feature alignment and information aggregation across modalities [18,19]. Despite their effectiveness, attention-based methods often incur substantial computational and memory costs when applied to high-resolution feature maps. Moreover, many existing approaches adopt homogeneous attention designs, lacking differentiated mechanisms to separately address modality-common alignment and modality-specific noise suppression.

Gating-based fusion employs learnable gating units to dynamically regulate the contribution of multimodal features [20,21]. By adaptively adjusting feature weights according to modality reliability or relevance, gating mechanisms alleviate modality imbalance and dominance issues. Owing to their low parameter count and computational efficiency, gating-based fusion methods are particularly suitable for adaptive multimodal integration in scenarios with varying modality quality.

Beyond these mainstream categories, several more advanced fusion mechanisms have also been explored. Tensor- or bilinear-pooling-based fusion captures high-order cross-modal interactions [22], but suffers from high computational and memory costs. Graph neural network (GNN)-based fusion leverages graph structures to facilitate cross-modality information propagation [23], but its performance heavily depends on graph construction and suffers from low efficiency in large-scale inference. Contrastive learning-based fusion aligns modalities by maximizing cross-modal consistency among positive samples while separating negative samples [24], showing strong performance in representation learning but requiring careful negative sampling and loss design. Generative model-based fusion explicitly models joint multimodal distributions [25], enabling cross-modal completion and generalization, yet often encounters training instability and high computational overhead.

### 2.3. Motivation

Existing RGB–IR multimodal fusion methods mainly explore the problem from two perspectives: fusion stage and fusion mechanism. Most approaches tend to treat features from different modalities as a whole and perform unified modeling and fusion. However, in real-world complex scenarios, modality-complementarity and noise interference often coexist, where noise primarily originates from unstable responses of modality-specific information under illumination variations, occlusions, or imaging discrepancies. Without explicitly distinguishing the intrinsic attributes of multimodal features, unreliable modality-specific characteristics may be indiscriminately introduced into the fused representations, regardless of the fusion stage or the fusion operator employed, thereby degrading discriminative performance.

**Figure 3:**
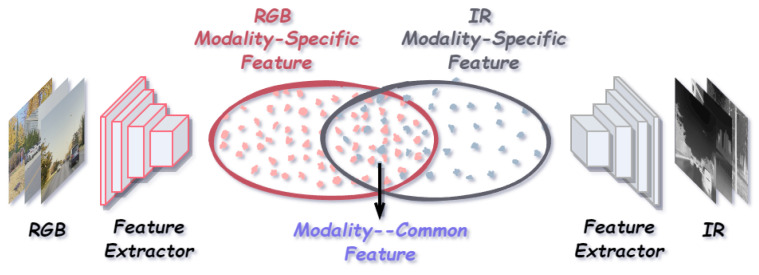
Illustration of modality-specific and modality-common feature decoupling.

Motivated by this observation, we argue that multimodal features should first be structurally decoupled into modality-common features and modality-specific features. The former capture cross-modal consistent and semantically stable shared information, while the latter encode modality-exclusive cues with complementary potential but uneven reliability. A conceptual illustration of this feature decoupling paradigm is presented in Figure 3. On this basis, differentiated enhancement strategies are required: modality-common features should be reinforced through cross-modal alignment and consistency modeling, whereas noise interference within modality-specific features should be suppressed to enable the selective utilization of complementary information. Finally, the enhanced modality-common features can serve as a semantic foundation to guide the adaptive fusion of modality-specific information, thereby achieving robust and effective cross-modal feature integration across diverse scenarios.

## 3. Proposed Method

This section presents the proposed DDAF-Net. We first introduce the overall network architecture. Subsequently, we provide a detailed description of the DAFM, which serves as the core component of our framework. Finally, the loss functions utilized for training are formulated.

### 3.1. Overview of DDAF-Net

Problem Definition. The goal of this study is to design an efficient multimodal fusion function F for object detection, enabling optimal integration of complementary information from heterogeneous modalities.

Given a pair of registered RGB and IR images, $I_{rgb}\in R^{3\times H_{0}\times W_{0}}$ and $I_{ir}\in R^{3\times H_{0}\times W_{0}}$, the task aims to predict a set of bounding boxes $B=\{b_{1},b_{2},\ldots ,b_{N}\}$ and their corresponding class labels $C=\{c_{1},c_{2},\ldots ,c_{N}\}$. DDAF-Net learns a robust fused representation $F_{fused}$ that effectively combines complementary cues while suppressing modality-specific noise. This process can be formalized as:

(1) $$\left(B,C\right)=D(H\left(F_{fused}\right)),$$

where $F_{fused}=F\left(I_{rgb},I_{ir}\right)$, H denotes the detection head, and D represents the decoding prediction module.

Formulation of Decoupled Representation. Motivated by the analysis in Section 2.3, we formulate the intermediate feature representations of RGB and IR modalities as the superposition of modality-common features, modality-specific features, and modality-dependent noise:

(2) $$\begin{array}{cc} F_{rgb} & =F^{c}+F_{rgb}^{s}+\epsilon_{rgb},\\ F_{ir} & =F^{c}+F_{ir}^{s}+\epsilon_{ir}, \end{array}$$

where $F^{c}$ denotes modality-common features shared across modalities, capturing semantically consistent information, $F_{m}^{s}$ represents modality-specific features with complementary potential, and $\epsilon_{m}$ corresponds to modality-dependent noise.

This formulation explicitly distinguishes cross-modal consistent semantics from modality-specific information, providing a principled basis for differentiated feature enhancement and guided fusion in the proposed framework.

**Figure 4:**
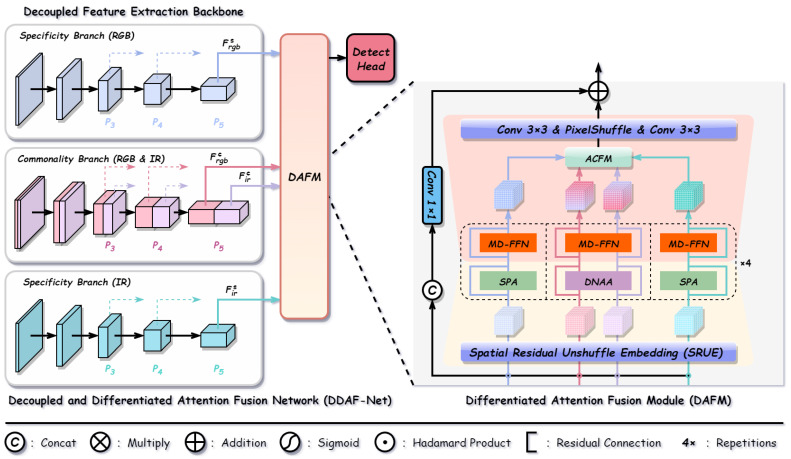
Overall architecture of the proposed DDAF-Net.

Network Architecture. As illustrated in Figure 4, the proposed DDAF-Net consists of two key components: the Decoupled Feature Extraction Backbone and the DAFM.

Following the decoupled representation formulation, the backbone adopts a three-branch structure based on a Siamese network architecture, extracting features at multi-scale pyramid levels (P3, P4, and P5). Specifically, two independent modality-specific branches are employed to capture the unique characteristics of each modality ($F_{rgb}^{s}$ and $F_{ir}^{s}$). Simultaneously, a parallel modality-common branch adopts a weight-sharing strategy to jointly model RGB and IR inputs, extracting cross-modal invariant common features ($F_{rgb}^{c}$ and $F_{ir}^{c}$). Consequently, at each scale, the backbone generates a decoupled feature quadruple $\left[F_{rgb}^{s},F_{rgb}^{c},F_{ir}^{c},F_{ir}^{s}\right]$, serving as the input for the subsequent fusion stage.

The core fusion process is executed by the DAFM. Its structure is illustrated in the zoomed-in view of Figure 4. Upon entering this module, the four feature maps are first projected via SRUE, which achieves lossless downsampling while preserving global semantic context. Subsequently, differentiated attention mechanisms are applied for feature processing: modality-specific features ($F_{rgb}^{s}$, $F_{ir}^{s}$) are refined by SPA to suppress noise and highlight complementary details, whereas modality-common features ($F_{rgb}^{c}$, $F_{ir}^{c}$) are modeled by the DNAA to facilitate effective modal alignment and enhance semantic consistency. Following this differentiated processing, all features are further enhanced by the MD-FFN, which reinforces local representations and information interaction across the channel, height, and width dimensions. Next, the enhanced features are aggregated via the ACFM, which utilizes modality-common features as a semantic anchor to dynamically weight and fuse complementary information from modality-specific features. Finally, the fused features undergo spatial upsampling to restore the original resolution and are element-wise added to the residual branch to generate the final module output. To promote deep feature interaction, the DAFM is stacked *N* times (default N=4) with residual connections.

Finally, the fused features are fed into the detection head H to predict object categories and regress bounding box coordinates.

### 3.2. Differentiated Attention Fusion Module (DAFM)

To effectively enhance and fuse decoupled modality-common and modality-specific features, we design the DAFM as the core fusion component of the network.

#### 3.2.1. Spatial Residual Unshuffle Embedding (SRUE)

**Figure 5:**
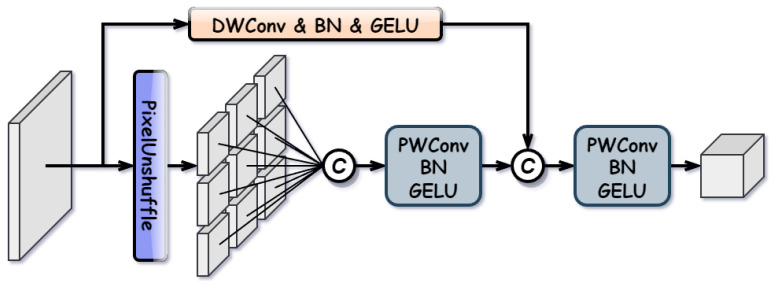
Structure of Spatial Residual Unshuffle Embedding (SRUE).

Standard downsampling operations, such as max pooling or strided convolution, are prone to losing fine-grained spatial information, whereas processing high-resolution features imposes significant computational burdens on attention mechanisms. To address this, we propose SRUE, which achieves lossless feature projection by preserving spatial details and global semantics during downsampling. The structure of SRUE is illustrated in Figure 5.

Specifically, the SRUE processes the input feature $X\in R^{C\times H\times W}$ through two parallel branches. The main branch rearranges spatial pixels into the channel dimension and adjusts the channels via pointwise convolution to retain local spatial information, formulated as:

(3) $$\begin{array}{cc} X_{main} & =\sigma (\mathrm{BN}\left({\mathrm{Conv}}_{pw}\left(\mathrm{PU}\left(X\right)\right)\right)),\\ \; & =F_{proj}\left(\mathrm{PU}\left(X\right)\right), \end{array}$$

where PU(·) denotes the pixel unshuffle operation with a downsampling factor *s*, which reshapes the tensor from C×H×W to $Cs^{2}\times \frac{H}{s}\times \frac{W}{s}$. ${\mathrm{Conv}}_{pw}$, BN, and σ represent pointwise convolution, Batch Normalization, and the activation function, respectively.

The residual branch utilizes a depthwise convolution with stride *s* and kernel size k=2s−1 to preserve contextual semantic information, formulated as:

(4) $$X_{res}=F_{proj}\left(\sigma \left(\mathrm{BN}\left({\mathrm{Conv}}_{dw}\left(X\right)\right)\right)\right),$$

where ${\mathrm{Conv}}_{dw}$ denotes depthwise convolution, and $F_{proj}$ aligns the channel dimensions with $X_{main}$ when necessary.

Finally, the lossless features from the main branch and the context-aware features from the residual branch are concatenated and fused to generate the output embedding $X_{out}$:

(5) $$X_{out}=F_{proj}\left(\left[X_{main},X_{res}\right]\right),$$

where [·,·] denotes concatenation along the channel dimension.

#### 3.2.2. Dual-Norm Alignment Attention (DNAA)

**Figure 6:**
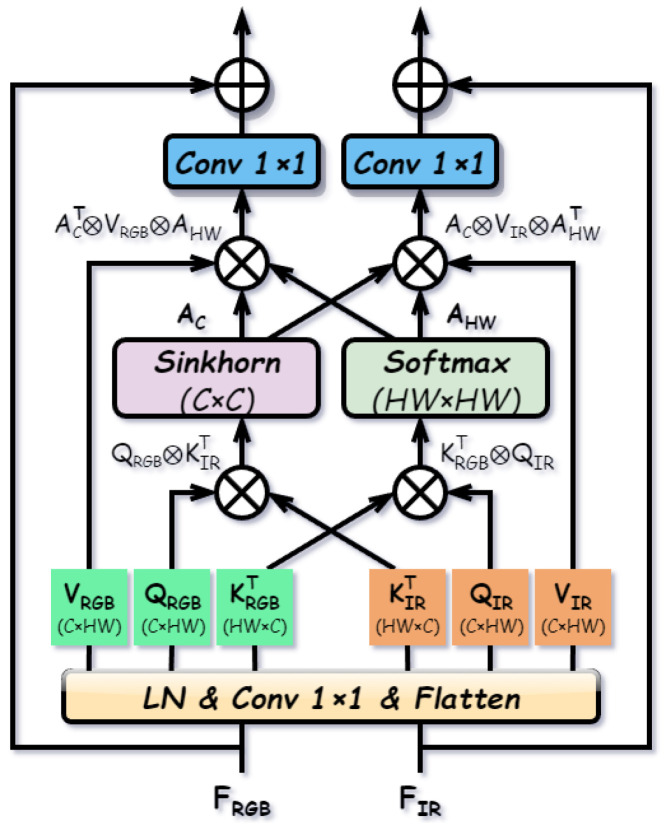
Structure of the Dual-Norm Alignment Attention (DNAA).

Although the Siamese backbone extracts modality-common features, inherent discrepancies in sensor imaging mechanisms and viewpoints inevitably lead to spatial and semantic misalignment between RGB and IR representations. To address this, we propose DNAA, which explicitly establishes cross-modal correspondences from both channel and spatial perspectives to enhance the semantic consistency of modality-common features. The structure of DNAA is illustrated in Figure 6.

Specifically, given the modality-common features $F_{rgb}^{c},F_{ir}^{c}\in R^{C\times H\times W}$, DNAA first applies Group Normalization and concatenates the features for joint projection. The query, key, and value matrices are generated via a unified linear projection and subsequent splitting, formulated as:

(6) $$\begin{array}{cc} \; & \left[Q_{rgb},K_{rgb},V_{rgb},Q_{ir},K_{ir},V_{ir}\right]\\ =\; & \mathrm{Split}({\mathrm{Conv}}_{1\times 1}\left(\left[\mathrm{GN}\left(F_{rgb}^{c}\right),\mathrm{GN}\left(F_{ir}^{c}\right)\right]\right)), \end{array}$$

where GN denotes Group Normalization. The generated matrices are flattened to $R^{C\times N}$, where N=H×W.

To align cross-modal semantic distributions, we introduce a channel alignment map $A_{c}\in R^{C\times C}$. Leveraging the intrinsic semantic consistency of modality-common features, we employ Sinkhorn iterations to enforce a doubly stochastic constraint, establishing a strict bijective mapping for robust global alignment. Conversely, for the spatial alignment map $A_{hw}\in R^{N\times N}$, the underlying correspondence is inherently non-bijective due to occlusion and parallax. Enforcing a strict Sinkhorn constraint here would erroneously force matches for occluded regions and incur prohibitive quadratic computational costs ($O({\left(HW\right)}^{2})$). Instead, we adopt Softmax normalization, which naturally accommodates asymmetric and multi-to-one relationships, allowing the suppression of irrelevant regions. This differentiated process is defined as:

(7) $$\begin{array}{cc} A_{c} & =\mathrm{Sinkhorn}\left(\frac{Q_{rgb}K_{ir}^{T}}{\sqrt{N}}\right),\\ A_{hw} & =\mathrm{Softmax}\left(\frac{K_{rgb}^{T}Q_{ir}}{\sqrt{C}}\right), \end{array}$$

where Sinkhorn(·) represents the iterative normalization process along both dimensions.

Finally, feature enhancement is achieved by jointly applying the channel and spatial alignment matrices to the value matrices. The aligned features are projected and added to the original inputs via residual connections:

(8) $$\begin{array}{cc} {\tilde{F}}_{rgb}^{c} & =F_{rgb}^{c}+{\mathrm{Conv}}_{1\times 1}\left(A_{c}^{T}V_{rgb}A_{hw}\right),\\ {\tilde{F}}_{ir}^{c} & =F_{ir}^{c}+{\mathrm{Conv}}_{1\times 1}\left(A_{c}V_{ir}A_{hw}^{T}\right). \end{array}$$

The enhanced features are then reshaped back to $R^{C\times H\times W}$. By aligning features specifically along both channel and spatial dimensions, DNAA effectively mitigates cross-modal discrepancies.

#### 3.2.3. Sparse Purification Attention (SPA)

**Figure 7:**
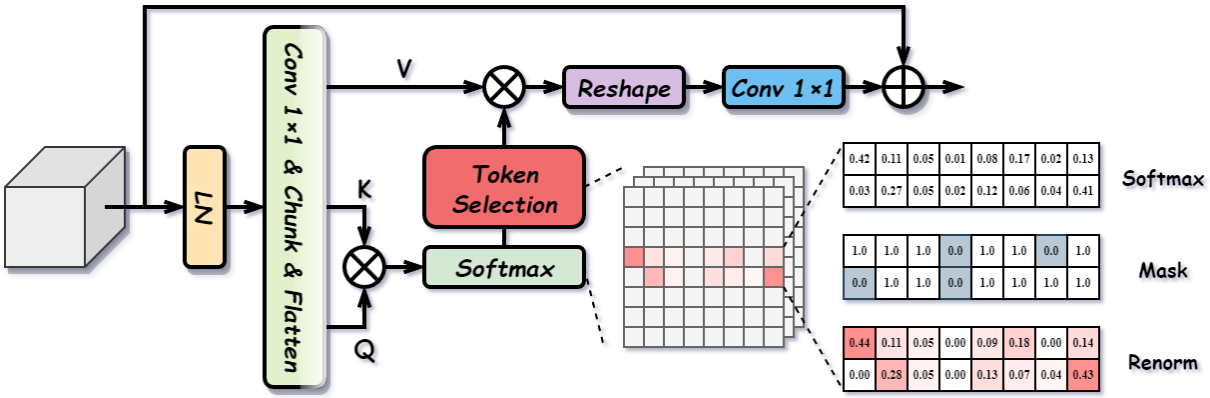
Structure of Sparse Purification Attention (SPA).

Modality-specific features, though rich in complementary information, are frequently contaminated by modality-dependent noise. Standard dense attention mechanisms, which aggregate features indiscriminately across all spatial positions, risk propagating or even amplifying this noise. To address this, we propose SPA. By leveraging sparsity constraints to filter out non-salient responses, SPA effectively suppresses noise while preserving critical complementary information. The structure of SPA is illustrated in Figure 7.

Specifically, for a given modality-specific feature $X\in R^{C\times H\times W}$ (representing either $F_{rgb}^{s}$ or $F_{ir}^{s}$), SPA first applies Group Normalization followed by linear projections to generate query (*Q*), key (*K*), and value (*V*) tensors. These are then reshaped for multi-head attention computation:

(9) $$\begin{array}{cc} Q,K,V & =R(\mathrm{Split}\left({\mathrm{Conv}}_{1\times 1}\left(\mathrm{GN}\left(X\right)\right)\right)),\\ A & =\mathrm{Softmax}\left(\frac{QK^{T}}{\sqrt{d_{k}}}\right), \end{array}$$

where R represents the reshaping operation for *h* heads, $d_{k}$ is the head dimension, and $A\in R^{B\times h\times N\times N}$ is the initial attention map.

To eliminate noise interference, we introduce a Top-*k* Purification mechanism. For each query position, only the top-*k* most relevant keys are retained. The attention map is then renormalized to ensure the weights sum to one:

(10) $$\begin{array}{cc} M_{i,j} & =\left\{\begin{array}{cc} 1, & \mathrm{if}\;A_{i,j}\in \mathrm{Top}\text{-}k\left(A_{i,\cdot }\right)\\ 0, & \mathrm{otherwise} \end{array}\right.,\\ {\hat{A}}_{i,j} & =\frac{A_{i,j}\cdot M_{i,j}}{\sum_{l}A_{i,l}\cdot M_{i,l}+\epsilon }, \end{array}$$

where M is the binary mask derived from the top-*k* indices determined by a retention ratio *r* (i.e., k=⌊N×r⌋), and ϵ is a small constant for numerical stability.

Finally, the purified attention map $\hat{A}$ aggregates the value features, and the output is projected back to the original dimension and added to the input via a residual connection:

(11) $$X_{out}=X+{\mathrm{Conv}}_{1\times 1}\left(R^{-1}\left(\hat{A}V\right)\right),$$

where $R^{-1}$ reshapes the multi-head output back to $R^{C\times H\times W}$. SPA effectively purifies modality-specific features, ensuring that only robust and salient complementary information is propagated.

#### 3.2.4. Multi-Dimensional Feed-Forward Network (MD-FFN)

**Figure 8:**
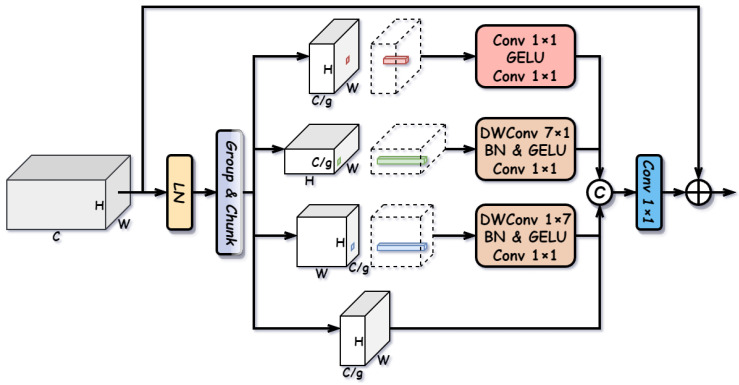
Structure of the Multi-Dimensional Feed-Forward Network (MD-FFN).

Standard feed-forward networks typically perform pixel-wise operations, focusing solely on channel mixing while neglecting local spatial contexts. Although the preceding attention modules capture global or sparse dependencies, enhancing local feature interactions along different geometric dimensions is crucial for refining object boundaries and shapes. To achieve this efficiently, we propose the MD-FFN. By decoupling feature processing into channel, height, and width dimensions, the MD-FFN captures multi-scale anisotropic contexts with minimal computational overhead. The structure of the MD-FFN is illustrated in Figure 8.

Specifically, given an input feature $X\in R^{C\times H\times W}$, the MD-FFN first applies Layer Normalization and splits the feature into four parts along the channel dimension, $\left\{X_{c},X_{h},X_{w},X_{id}\right\}$, where each part has $\frac{C}{4}$ channels. These parts are processed by four parallel branches to extract complementary contextual information:

(1) Channel Mixing Branch: This branch follows the standard FFN design to model inter-channel dependencies using pointwise convolutions:

(12) $$Y_{c}={\mathrm{Conv}}_{pw}\left(\sigma \left({\mathrm{Conv}}_{pw}\left(X_{c}\right)\right)\right).$$

(2) Height and Width Mixing Branches: To capture anisotropic spatial contexts without the high cost of large dense kernels, we employ strip depthwise convolutions. The height branch uses a 7×1 kernel to aggregate vertical context, while the width branch uses a 1×7 kernel for horizontal context:

(13) $$\begin{array}{cc} Y_{h} & ={\mathrm{Conv}}_{pw}\left(\sigma \left(\mathrm{BN}\left({\mathrm{Conv}}_{dw}^{7\times 1}\left(X_{h}\right)\right)\right)\right),\\ Y_{w} & ={\mathrm{Conv}}_{pw}\left(\sigma \left(\mathrm{BN}\left({\mathrm{Conv}}_{dw}^{1\times 7}\left(X_{w}\right)\right)\right)\right). \end{array}$$

(3) Identity Branch: The fourth part $X_{id}$ remains unchanged to preserve original feature information.

Finally, the outputs from all branches are concatenated and fused via a linear projection, followed by a residual connection to the original input:

(14) $$X_{out}=X+{\mathrm{Conv}}_{1\times 1}\left(\left[Y_{c},Y_{h},Y_{w},X_{id}\right]\right).$$

By explicitly modeling interactions along the channel, height, and width axes, the MD-FFN significantly enhances the local representation capability of the network.

#### 3.2.5. Adaptive Complementary Fusion Module (ACFM)

**Figure 9:**
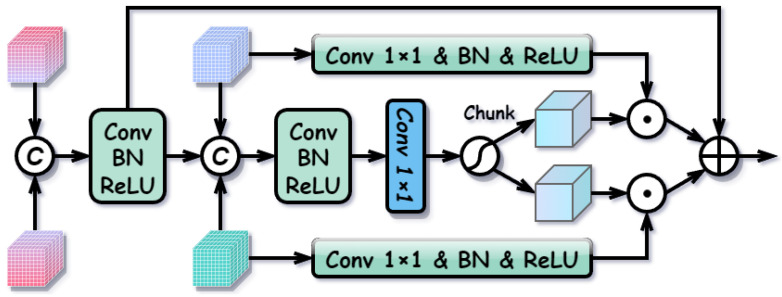
Structure of the Adaptive Complementary Fusion Module (ACFM).

After the differentiated enhancement, the network obtains aligned modality-common features and purified modality-specific features. Simple fusion strategies, such as element-wise addition or concatenation, treat these components indiscriminately, lacking flexibility to adapt to dynamic scene variations (e.g., RGB failure in total darkness). To address this, we propose the ACFM. The ACFM utilizes the stable modality-common features as a semantic anchor to dynamically regulate the incorporation of complementary modality-specific information. The structure of the ACFM is illustrated in Figure 9.

Specifically, the ACFM takes four feature maps as input: two aligned common features ($F_{rgb}^{c},F_{ir}^{c}$) and two purified specific features ($F_{rgb}^{s},F_{ir}^{s}$). First, we construct a robust semantic baseline $F_{base}$ by fusing the modality-common features, which contain consistent geometric and semantic cues:

(15) $$F_{base}=\sigma \left(\mathrm{BN}\left({\mathrm{Conv}}_{3\times 3}\left(\left[F_{rgb}^{c},F_{ir}^{c}\right]\right)\right)\right).$$

Next, to determine the reliability of modality-specific information, we generate dynamic gating weights. The base feature is concatenated with both modality-specific features to form a comprehensive context representation, which is then processed by a gating network:

(16) $$\begin{array}{cc} G_{total} & =\mathrm{GateNet}(\left[F_{base},F_{rgb}^{s},F_{ir}^{s}\right]),\\ W_{rgb},W_{ir} & =\mathrm{Split}(G_{total}), \end{array}$$

where GateNet consists of a 3×3 convolution followed by a 1×1 projection and a Sigmoid function, producing weights $W_{rgb},W_{ir}\in \left[0,1\right]$ that represent the importance of RGB- and IR-specific details, respectively.

Finally, the modality-specific features are transformed to align with the baseline space and adaptively aggregated based on the generated weights:

(17) $$\begin{array}{cc} {\tilde{F}}_{rgb}^{s} & =\sigma (\mathrm{BN}\left({\mathrm{Conv}}_{1\times 1}\left(F_{rgb}^{s}\right)\right)),\\ {\tilde{F}}_{ir}^{s} & =\sigma (\mathrm{BN}\left({\mathrm{Conv}}_{1\times 1}\left(F_{ir}^{s}\right)\right)),\\ F_{fused} & =F_{base}+W_{rgb}⊙{\tilde{F}}_{rgb}^{s}+W_{ir}⊙{\tilde{F}}_{ir}^{s}, \end{array}$$

where ⊙ denotes element-wise multiplication. By anchoring the fusion on stable common semantics and adaptively selecting complementary details, the ACFM ensures robust feature integration across varying environmental conditions.

### 3.3. Loss Function

The training of DDAF-Net is supervised by a composite objective function that simultaneously optimizes object detection performance and enforces the structural decoupling of multimodal features. The total loss $L_{total}$ is formulated as:

(18) $$L_{total}=L_{det}+\lambda_{orth}L_{orth},$$

where $L_{det}$ represents the task-specific detection loss, and $L_{orth}$ denotes the orthogonality constraint for feature decoupling. The hyperparameter $\lambda_{orth}$ balances the trade-off between detection accuracy and feature independence, which is set to 0.1 in our experiments. Importantly, the orthogonality constraint is introduced as a soft regularization term rather than a primary optimization objective. It does not dominate the training process, but instead gently encourages feature independence without compromising the learning of the main detection task.

Detection Loss. Following standard object detection paradigms, the detection head is optimized using a weighted sum of classification, bounding box regression, and objectness losses:

(19) $$L_{det}=0.5L_{cls}+7.5L_{box}+1.5L_{obj},$$

where $L_{cls}$ denotes the classification loss, $L_{box}$ represents the bounding box regression loss, and $L_{obj}$ indicates the objectness loss for foreground-background confidence.

Orthogonality Loss. To validate the proposed decoupling paradigm, it is crucial to ensure that the modality-common features ($F^{c}$) and modality-specific features ($F^{s}$) encode distinct semantic information rather than redundant cues. To achieve this, we introduce an orthogonality constraint that minimizes the cosine similarity between the two representations. For each modality m∈{rgb,ir} across all pyramid levels, the loss is defined as:

(20) $$L_{orth}=\sum_{m,l}\left|\frac{\mathrm{vec}\left(F_{m,l}^{c}\right)\cdot \mathrm{vec}\left(F_{m,l}^{s}\right)}{∥\mathrm{vec}\left(F_{m,l}^{c}\right){∥}_{2}\cdot {∥\mathrm{vec}\left(F_{m,l}^{s}\right)∥}_{2}+\epsilon }\right|,$$

where vec(·) flattens the feature map into a vector, and ${∥\cdot ∥}_{2}$ denotes the $L_{2}$ norm. By minimizing $L_{orth}$, the network is penalized for feature correlation, thereby forcing the specific branch to capture complementary details exclusive to the modality, separate from the shared semantics in the common branch.

## 4. Experiments

### 4.1. Datasets and Evaluation Metrics

To comprehensively evaluate the effectiveness of the proposed method, experiments were conducted on three representative and complementary multimodal datasets: FLIR [26], LLVIP [27], and M^3^FD [28].

The FLIR dataset is designed for multimodal object detection and covers both daytime and nighttime scenes. Since the original version suffers from significant misalignment between modalities, this study adopts the aligned FLIR-Aligned version, which contains 5142 pairs of RGB–IR images (4129 for training and 1013 for testing) with a resolution of 640 × 512. It includes classes such as Person, Car, Dog, and Bicycle. The major challenges of this dataset lie in the large domain gap between different time periods and the need for effective exploitation of infrared cues.

The LLVIP dataset focuses on pedestrian detection under low-light conditions. It contains 15,488 pairs of RGB–IR images (12,025 for training and 3463 for testing) with a resolution of 1280 × 1024. Its only annotated class is Person. The main difficulty lies in the severe degradation of the RGB modality under low illumination, which places higher demands on cross-modality complementarity.

The M^3^FD dataset consists of 4200 pairs of RGB–IR images (resolution 1024 × 768), annotated with over 34,000 instances across six classes (People, Car, Bus, Motorcycle, Truck, and Lamp). Since there is no official split, an 80%/20% division is adopted for training and testing, respectively. M^3^FD features diverse categories and scenes but suffers from certain misalignment issues, posing challenges for robust detection across modalities.

For evaluation, the mean Average Precision (mAP) following the COCO protocol is employed as the primary metric, including the following:

- mAP@0.5 (AP_50_): average precision at IoU threshold 0.5.
- mAP@0.75 (AP_75_): average precision at IoU threshold 0.75.
- mAP@0.5:0.95 (mAP): average precision averaged over IoU thresholds from 0.5 to 0.95, reflecting detection performance under varying strictness.

In addition, to assess practical efficiency, the inference time and model parameters are also reported, providing a balanced evaluation of accuracy and computational cost.

### 4.2. Implementation Details

The proposed DDAF-Net is implemented based on the Ultralytics YOLOv8 framework. During training, all images are resized to 640 × 640, and standard data augmentation techniques such as Mosaic and RandAugment are applied. The optimizer is AdamW with an initial learning rate of 0.001, momentum of 0.937, and weight decay of 0.0005. The total number of training epochs is 300, with a batch size of 16. To ensure reproducibility, the deterministic mode is enabled and random seeds are fixed.

Experiments are conducted on four NVIDIA A30 GPUs (24 GB each) using Python 3.8.20, PyTorch 2.3.1, and Ultralytics v8.3.146.

### 4.3. Quantitative Comparisons

To thoroughly evaluate the proposed DDAF-Net for multimodal sensor-based object detection, this section compares it with representative infrared–visible fusion detectors in terms of overall accuracy, computational efficiency, and per-class performance.

#### 4.3.1. Comparison with Representative Methods

To validate the detection performance of DDAF-Net under different scenarios, we conduct systematic comparisons on the FLIR-Aligned and LLVIP datasets against multiple baseline single-modality detectors and state-of-the-art multimodal fusion approaches. Table 1 reports the results on AP_50_, AP_75_, and mAP metrics.

These results indicate that single-modality detectors are limited by environmental conditions, whereas IR offers better stability than RGB. Multimodal fusion methods significantly enhance detection by leveraging complementary information. DDAF-Net achieves state-of-the-art performance on both datasets, recording 50.5% mAP on FLIR-Aligned and 68.7% mAP on LLVIP, outperforming the latest Fusion-Mamba (TMM’25). Notably, DDAF-Net exhibits a substantial lead in the stricter AP_75_ metric (52.6% and 78.2%, respectively), verifying its superior capability in precise object localization and feature discrimination.

#### 4.3.2. Inference Time and Computational Efficiency

**Figure 10:**
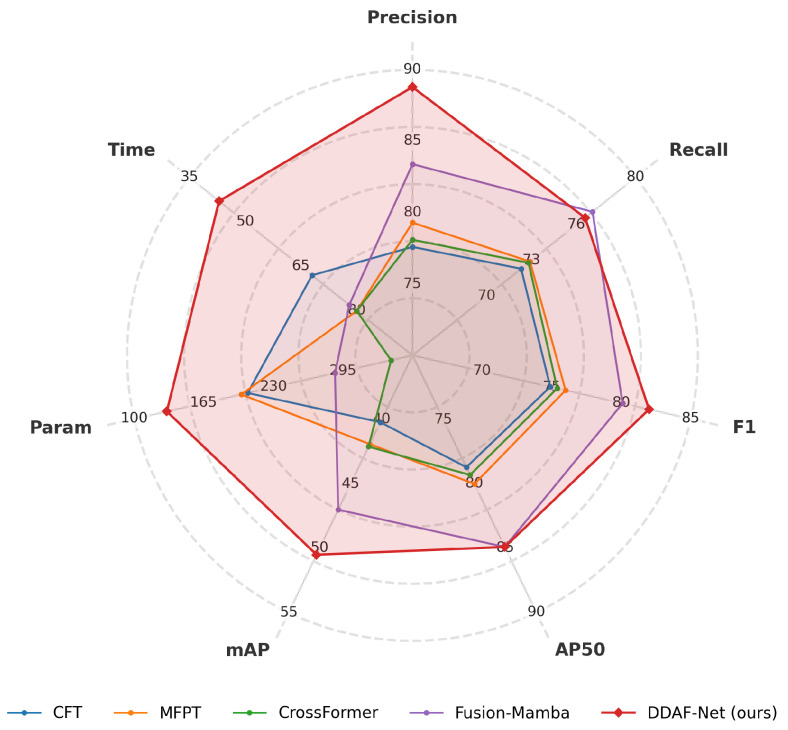
Comprehensive radar chart of DDAF-Net’s performance in terms of accuracy and computational efficiency. Param. and Time are inverted for visualization (smaller values correspond to larger radii). The compared methods include CFT [35], MFPT [41], CrossFormer [32], and Fusion-Mamba [39].

In real-world applications, balancing computational complexity with inference efficiency is crucial for model practicality. To assess this, we compare DDAF-Net against representative methods on the FLIR-Aligned dataset across accuracy metrics (precision, recall, F1, AP_50_, mAP) and efficiency indicators (parameters, time). The comprehensive results are illustrated in the radar chart in Figure 10.

DDAF-Net exhibits the most robust overall performance, covering the largest area in the radar chart. Regarding detection accuracy, it achieves the highest precision (88.78%), F1-score (81.98%), and mAP (50.53%), while matching the top performance in AP_50_ (84.89%). Crucially, in terms of efficiency, DDAF-Net demonstrates a significant advantage: it requires only 130.5 M parameters and 43 ms inference time, making it drastically lighter and faster than the state-of-the-art Fusion-Mamba (287.6 M, 78 ms) and CrossFormer (340.0 M, 80 ms). This comparison confirms that DDAF-Net offers an optimal trade-off between high-precision detection and low computational cost, validating its potential for efficient real-time deployment.

#### 4.3.3. Per-Class Performance Analysis

To further evaluate DDAF-Net’s capability in complex multi-category scenes, we conduct comparisons with recent multimodal fusion detectors on the M^3^FD dataset. Table 2 summarizes the per-class detection results using different backbones.

The results demonstrate that DDAF-Net establishes new state-of-the-art performance. Equipped with the CSPDarknet53v8 backbone, it achieves 90.6% AP_50_ and 63.5% mAP, surpassing the latest Fusion-Mamba by 2.6% and 1.6%, respectively. Even with the lighter v5 backbone, DDAF-Net (88.1% AP_50_) remains competitive, outperforming all other methods using the same backbone architecture.

In terms of category-specific performance, DDAF-Net shows exceptional strength in detecting small and distinct targets. Notably, in the challenging People category, it reaches 88.5%, significantly outperforming Fusion-Mamba (84.3%) by 4.2%. Furthermore, it dominates large-scale vehicle detection, securing the best scores in Bus (96.8%) and Truck (90.7%), while maintaining top-tier performance in other categories like Motorcycle (86.0%) and Car (94.2%). This balanced performance across diverse categories validates the model’s strong inter-class consistency and its ability to effectively leverage cross-modality features for both fine-grained and large-scale objects.

### 4.4. Qualitative Analysis

**Figure 11:**
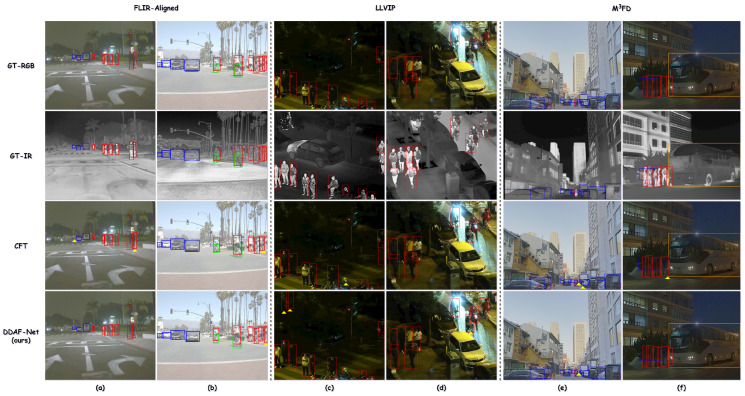
Representative results on the FLIR-Aligned (**a**,**b**), LLVIP (**c**,**d**), and M^3^FD (**e**,**f**) datasets. Yellow triangles indicate false detections that do not match the ground truth. The reader can zoom in for details. Category annotations: Car (blue), Person (red), Bicycle (green), Motorcycle (purple), and Bus (orange).

To intuitively demonstrate the detection performance of DDAF-Net, representative samples were selected from the FLIR-Aligned (a, b), LLVIP (c, d), and M^3^FD (e, f) datasets for visualization, as illustrated in Figure 11. These samples cover various challenging scenarios, including low illumination, strong lighting, occlusion, dense crowds, and small objects. All visualizations were performed under a confidence threshold of 0.5.

DDAF-Net can accurately localize and recognize objects even under severe occlusion, dense scenes, or cases where targets are hardly visible in the RGB modality. In contrast, the CFT method tends to suffer from both missed and false detections under such conditions. Particularly in Figure 11b–d, DDAF-Net not only detects most ground-truth objects precisely but also identifies potential targets that other methods overlook, demonstrating its superiority in cross-modal feature complementarity. Moreover, DDAF-Net shows a clear advantage in boundary precision and small-object detection (see Figure 11e). These qualitative results further validate the robustness and generalization capability of the proposed model under diverse and complex scenes.

### 4.5. Ablation Studies

In this section, we perform comprehensive ablation studies on the M^3^FD dataset to validate the effectiveness of the proposed framework and the necessity of each specific component.

To investigate the validity of the proposed “decoupling–enhancement–fusion” paradigm, we constructed two control groups for comparison with our DDAF-Net:

1. Two-Stream: a standard uncoupled architecture where RGB and IR branches are independent (no weight sharing) and fused via simple concatenation and convolution.
2. Baseline: the proposed Decoupled Feature Extraction Backbone combined with naive fusion (concatenation + convolution), without the DAFM.

The results are reported in Table 3. Interestingly, the Baseline (54.7% mAP) performs slightly worse than the traditional Two-Stream architecture (56.1% mAP). This phenomenon supports our analysis in Section 1: simply decoupling features into modality-common and modality-specific parts forces them into distinct semantic subspaces. Without explicit alignment and enhancement mechanisms, naive concatenation fails to reconcile the spatial misalignment in common features or filter the noise in specific features, leading to suboptimal feature integration.

However, when the DAFM is introduced, the performance improves dramatically, jumping from 54.7% to 63.5% mAP. This substantial gain (+8.8% mAP) confirms that the decoupled features possess high potential, but their value can only be fully unlocked through differentiated processing—specifically, aligning the common semantics and purifying the specific details.

To verify that the performance gains stem from our specific differentiated designs (DNAA, SPA, ACFM) rather than the mere application of attention mechanisms, we conducted an internal ablation of the DAFM. We replaced each proposed module with its generic counterpart to observe the impact:

- w/o DNAA: replaces DNAA with standard cross-attention.
- w/o SPA: replaces SPA with standard dense self-attention.
- w/o ACFM: replaces ACFM with feature concatenation.
- Generic Attn: replaces all three modules with their generic counterparts simultaneously.

The results are summarized in Table 4. Replacing all proposed components with generic attention and naive fusion yields 58.5% mAP, which is higher than the baseline (54.7%) due to the global receptive field of attention, yet still significantly inferior to the full model (63.5%), indicating that performance gains do not stem from attention mechanisms alone. Specifically, substituting SPA with dense self-attention causes the largest degradation (−3.0% mAP), confirming that modality-specific features contain substantial noise that is amplified by indiscriminate global aggregation, whereas SPA effectively purifies these features via sparsity constraints. Replacing DNAA with standard cross-attention results in a 2.3% mAP drop, as cross-attention lacks the explicit bijective alignment enforced by the Sinkhorn-based dual-normalization design, which is crucial for maintaining semantic consistency in modality-common features. Finally, removing the adaptive fusion mechanism leads to a 1.6% mAP decline, demonstrating that simple concatenation fails to handle dynamic modality reliability, while the ACFM leverages aligned common features as a semantic anchor to robustly fuse complementary information.

To further provide a detailed computational complexity analysis and evaluate the module-wise overhead, we conducted a hyperparameter sensitivity study on the number of stacked DAFM blocks (*N*). Table 5 reports the detection accuracy along with comprehensive complexity metrics, including parameters, network Layers, FLOPs, and inference speed (FPS).

As shown in Table 5, the proposed DAFM is highly parameter- and computation-efficient. Increasing the number of stacked blocks from 2 to 4 brings a significant performance gain of +1.1% mAP while only introducing a marginal overhead of 0.8 M parameters and 2.78 G FLOPs. This indicates that the core components of the DAFM (DNAA, SPA, and ACFM) focus on efficient feature recalibration rather than heavy channel transformations. However, when the stack deepens to N=8, the performance saturates (only a +0.2% mAP improvement), yet the inference speed drops drastically from 19.39 FPS to 8.82 FPS due to the sequential execution of over 3000 layers. Consequently, we adopt N=4 as the default setting to achieve the optimal trade-off between detection accuracy and computational efficiency.

**Figure 12:**
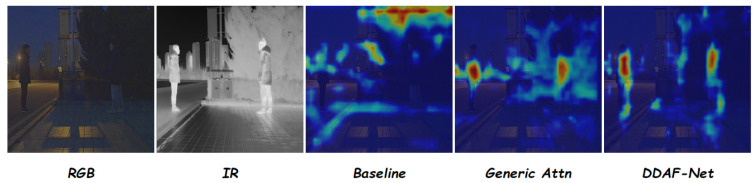
EigenCAM heatmaps for Baseline, Generic Attn, and DDAF-Net.

As shown in Figure 12, we further visualize the feature-level heatmaps using EigenCAM [54] for the Baseline, Generic Attn, and DDAF-Net. The heatmaps reveal that the Baseline model focuses on many irrelevant regions, while Generic Attn introduces noise due to its global aggregation. In contrast, DDAF-Net achieves much more concentrated attention on the salient areas, confirming that our proposed design not only improves performance but also enhances feature discrimination by focusing on the most informative regions.

## 5. Conclusions and Future Work

In this paper, we proposed DDAF-Net to address the persistent challenges of modality redundancy, noise interference, and spatial misalignment in RGB-IR object detection. By establishing an explicit “decoupling–enhancement–fusion” paradigm, our method effectively decomposes multimodal inputs into modality-common and modality-specific representations. Within the proposed DAFM, DNAA ensures robust semantic consistency across modalities, while SPA successfully filters modality-dependent noise to retain distinct complementary details. Furthermore, the ACFM utilizes the aligned common semantics as an anchor to dynamically guide the integration of these features. Extensive experiments on the FLIR-Aligned, LLVIP, and M^3^FD datasets demonstrate that DDAF-Net achieves state-of-the-art performance in both detection accuracy and inference efficiency, validating the superiority of the proposed differentiated modeling strategy.

Despite these promising results, several avenues for future research remain. First, while DDAF-Net achieves a favorable trade-off between accuracy and speed, deployment on strictly resource-constrained edge devices (e.g., UAVs or IoT sensors) may require further optimization techniques such as network quantization or distillation. Second, although DNAA handles mild spatial misalignment effectively, evaluating the model’s performance boundaries under extreme RGB-IR misalignment (e.g., large viewpoint offsets or resolution mismatches) and severe single-modality occlusion remains a critical challenge. Furthermore, our current framework operates under the assumption of registered image pairs. In real-world deployments, dynamic scenes or sensor calibration errors frequently result in completely unregistered pairs, which can significantly degrade fusion efficacy. To address these limitations, future research will explore integrating lightweight registration preprocessing steps or flow-based dense feature matching networks directly into the pipeline, enabling robust end-to-end detection on unaligned sensor data. Finally, the proposed decoupling paradigm is theoretically generic and could be extended to other heterogeneous sensor combinations, such as LiDAR-Camera or Event-RGB fusion, to further broaden the applicability of robust multimodal perception systems.

## Figures and Tables

**Table 1:** Performance comparison on the FLIR-Aligned and LLVIP datasets.

Methods	Pub.	Modality	FLIR-Aligned			LLVIP		
			AP_50_	AP_75_	mAP	AP_50_	AP_75_	mAP
Faster R-CNN	TPAMI’15	IR	73.4	34.2	37.9	92.6	48.8	50.7
YOLOv5	’20	IR	73.9	35.7	39.5	94.6	72.2	61.9
YOLOv8	’23	IR	72.9	34.8	38.3	95.2	72.5	62.1
Faster R-CNN	TPAMI’15	RGB	65.0	22.8	30.2	88.8	45.7	47.5
YOLOv5	’20	RGB	67.8	25.9	31.8	90.8	51.9	50.0
YOLOv8	’23	RGB	66.3	25.0	28.2	91.9	53.0	54.0
GAFF [29]	WACV’21	IR+RGB	72.9	32.9	37.5	94.0	60.2	55.8
ProbEn [30]	ECCV’22	IR+RGB	75.5	31.8	37.9	93.4	50.2	51.5
CSAA [31]	CVPR’23	IR+RGB	79.2	37.4	41.3	94.3	66.6	59.2
CrossFormer [32]	PRL’24	IR+RGB	79.3	38.5	42.1	97.4	*75.4*	65.1
RSDet [33]	arXiv’24	IR+RGB	83.9	40.1	43.8	95.8	70.4	61.3
Fusion-DETR [34]	’25	IR+RGB	81.5	–	44.3	96.4	–	64.6
CFT [35]	ArXiv’21	IR+RGB	78.7	35.5	40.2	*97.5*	72.9	63.6
YOLO-MS [36]	TCDS’23	IR+RGB	75.2	–	38.3	94.9	–	60.2
ICAFusion [37]	PR’24	IR+RGB	79.2	36.9	41.4	95.2	–	60.1
LRAF-Net [38]	TNNLS’24	IR+RGB	80.5	–	42.8	**97.9**	–	*66.3*
Fusion-Mamba [39]	TMM’25	IR+RGB	**84.9**	*45.9*	*47.0*	97.0	72.2	64.3
MS2Fusion [40]	Information Fusion’25	IR+RGB	83.3	–	40.3	97.5	–	65.5
DDAF-Net (ours)	–	IR+RGB	**84.9**	**52.6**	**50.5**	**97.9**	**78.2**	**68.7**

Note: The column “Pub.” denotes the publication venue of each method. The best scores are highlighted in **bold**, the second-best scores are *italicized*, and missing values are denoted by ‘–’.

**Table 2:** Per-class performance comparison on the M^3^FD dataset.

Methods	Backbone	AP_50_	mAP	Per-Class AP_50_					
				People	Car	Lamp	Motorcycle	Bus	Truck
DIDFuse [42]	CSPDarknet53v5	78.9	52.6	79.6	92.5	84.7	68.7	79.6	68.7
SDNet [43]	CSPDarknet53v5	79.0	52.9	79.4	92.3	84.1	67.4	81.4	69.3
RFNet [44]	CSPDarknet53v5	79.4	53.2	79.4	91.1	85.0	72.8	78.2	69.0
TarDAL [28]	CSPDarknet53v5	80.5	54.1	81.5	**94.8**	87.1	69.3	81.3	68.7
DetFusion [45]	CSPDarknet53v5	80.8	53.8	80.8	92.5	*87.8*	69.4	83.0	71.4
CDDFuse [5]	CSPDarknet53v5	81.1	54.3	81.6	92.5	86.9	71.6	82.6	71.5
IGNet [46]	CSPDarknet53v5	81.5	54.5	81.6	92.8	86.9	73.0	82.4	72.1
MMFN [47]	CSPDarknet53v5	86.2	57.4	83.0	93.2	87.6	73.7	92.1	87.4
EMMA [48]	CSPDarknet53v5	82.9	55.4	82.0	93.5	87.7	77.7	83.2	73.5
KCDNet [49]	CSPDarknet53v5	83.2	56.3	83.3	90.9	80.3	84.1	88.4	72.1
CRSIOD [50]	CSPDarknet53v5	84.0	57.2	78.4	92.2	80.5	73.9	93.3	85.5
SuperFusion [51]	E-ELAN	83.5	56.0	83.7	91.0	70.0	77.4	93.2	85.8
TSJNet [52]	E-ELAN	86.0	58.9	81.8	91.8	70.4	**86.8**	*95.7*	89.3
RI-YOLO [53]	CSPDarknet53v8	83.6	56.6	84.1	91.6	75.7	73.5	88.5	87.8
Fusion-Mamba [39]	CSPDarknet53v8	88.0	61.9	84.3	92.9	87.5	80.5	94.2	88.8
MS2Fusion [40]	CSPDarknet53v8	*89.4*	59.7	85.6	93.9	**90.8**	82.4	93.7	*89.9*
DDAF-Net (ours)	CSPDarknet53v5	88.1	*62.0*	*87.2*	92.9	86.5	82.2	91.9	86.9
	CSPDarknet53v8	**90.6**	**63.5**	**88.5**	*94.2*	87.5	*86.0*	**96.8**	**90.7**

Bold represents the largest value, and italics represent the second largest value.

**Table 3:** Analysis of the decoupling–fusion paradigm on M^3^FD.

Model Architecture	AP_50_	AP_75_	mAP
Two-Stream (Standard)	82.5	59.1	56.1
Baseline (Decoupled)	81.0	58.5	54.7
Baseline + DAFM (Ours)	90.6	68.0	63.5

**Table 4:** Ablation studies of the internal components of the DAFM on M^3^FD.

Method	Alignment (DNAA)	Purification (SPA)	Fusion (ACFM)	AP_50_	mAP
DDAF-Net	✓	✓	✓	90.6	63.5
w/o DNAA	→CA	✓	✓	88.4	61.2
w/o SPA	✓	→SA	✓	87.5	60.5
w/o ACFM	✓	✓	→Cat	89.1	61.9
Generic Attn	→CA	→SA	→Cat	85.2	58.5

Note: Arrows (→) indicate replacing the proposed module with a generic counterpart (CA: cross-attention, SA: self-attention, Cat: concatenation).

**Table 5:** Hyperparameter sensitivity and computational complexity analysis of stacked DAFM blocks on the M^3^FD dataset.

DAFM Blocks (*N*)	mAP	Params (M)	Layers	FLOPs (G)	FPS
2	62.4	129.7	1521	312.82	27.31
4 (Default)	63.5	130.5	2025	315.60	19.39
8	63.7	132.6	3033	321.24	8.82

## Data Availability

Publicly available datasets were analyzed in this study. The data, code, and models presented in this study are available at https://github.com/noob960205/DDAF-Net (accessed on 25 May 2025).

## Abbreviations

The following abbreviations are used in this manuscript:

RGB	Red–Green–Blue (Visible Light)
IR	Infrared
DDAF-Net	Decoupled and Differentiated Attention Fusion Network
DAFM	Differentiated Attention Fusion Module
DNAA	Dual-Norm Alignment Attention
SPA	Sparse Purification Attention
ACFM	Adaptive Complementary Fusion Module
mAP	mean Average Precision
IoU	Intersection over Union
